# Selecting the embryo with the highest implantation potential using developmental and morphometric scoring.

**DOI:** 10.1186/2049-3258-73-S1-P48

**Published:** 2015-09-17

**Authors:** Fang Chen, Sophie Debrock, Karen Peeraer, Thomas D'hooghe, Carl Spiessens

**Affiliations:** 1 University Hospitals Leuven, Leuven, Belgium; 2 Leuven University Fertility Center, Leuven, Belgium

## 

Embryo selection has been based on developmental and morphological characteristics. However, the presence of an important intra-and inter-observer variability in the microscopic evaluation of standard scoring system (SSS) has been reported. A golden standard for selection of embryos on day 3 is currently lacking. New technology using multilevel images combined with a computer-assisted scoring system (CASS) has the potential to overcome most of these disadvantages associated with the SSS.

The aim of this study was to construct a multivariable prediction model, with data mining approaches, and compare the predictive performance of models in SSS and CASS. A total of 871 single embryo transfers between 2008 and 2013 were included. Embryos were evaluated with two scoring systems: SSS and CASS. Prediction models were developed using multivariable logistic regression (LR) and multivariate adaptive regression splines (MARSplines). The prediction models were externally validated with a test set of 109 single transfers between January and June 2014. Results showed that prediction of implantation was superior using CASS when compared with SSS. With CASS, two final prediction model were obtained using LR and MARSplines, which showed moderate discriminative capacity (c-statistic 0.64 and 0.69 respectively) on validation data. The findings of this study show that combination of computer-assisted scoring system and machine learning based prediction method is a promising approach for in the selection of embryo with the highest implantation potential.

